# UK health researchers’ considerations of the environmental impacts of their data-intensive practices and its relevance to health inequities

**DOI:** 10.1186/s12910-023-00973-2

**Published:** 2023-10-27

**Authors:** Gabrielle Samuel

**Affiliations:** Department of Global Health and Social Medicine, King’s College London, London, Strand UK

**Keywords:** Environmental impacts, Data, Health, Research, Health inequities, Ethics

## Abstract

**Background:**

The health sector aims to improve health outcomes and access to healthcare. At the same time, the sector relies on unsustainable environmental practices that are increasingly recognised to be catastrophic threats to human health and health inequities. As such, a moral imperative exists for the sector to address these practices. While strides are currently underway to mitigate the environmental impacts of healthcare, less is known about how health researchers are addressing these issues, if at all.

**Methods:**

This paper uses an interview methodology to explore the attitudes of UK health researchers using data-intensive methodologies about the adverse environmental impacts of their practices, and how they view the importance of these considerations within wider health goals.

**Results:**

Interviews with 26 researchers showed that participants wanted to address the environmental and related health harms associated with their research and they reflected on how they could do so in alignment with their own research goals. However, when tensions emerged, their own research was prioritised. This was related to their own desires as researchers and driven by the broader socio-political context of their research endeavours.

**Conclusion:**

To help mitigate the environmental and health harms associated with data-intensive health research, the socio-political context of research culture must be addressed.

**Supplementary Information:**

The online version contains supplementary material available at 10.1186/s12910-023-00973-2.

## Introduction

The health sector aims to improve health outcomes and access to healthcare. At the same time, the sector relies on unsustainable environmental practices that are increasingly recognised to be catastrophic threats to human health and health inequities. The health sector contributes between 1% and 5% of various global environmental impacts, including greenhouse gas emissions, particulate matter, air pollutants, reactive nitrogen in water and scarce water use [1]. Impacts are country dependent, for instance, the United States healthcare industry emits nearly 8% of the country’s total greenhouse gas emissions, for Great Britain it is about 6%, with Mexico’s healthcare sector contributing approximately 3.3% to its nation’s emissions [2]. Healthcare is also a massive emitter of waste [3], much of which is plastic, with single-use plastic items (syringes, blood bags, tubing) saturating everyday medical practice across the globe [4]. Other environmental impacts include the use of water and the production of a variety of soil and water pollutants [1].

Healthcare research has its own specific effects on climate change and other adverse environmental harms [5–7]. For example, research laboratories generate more greenhouse gas emissions than the average household [5, 6]; bioscience research laboratories are estimated to consume 5.5 million tonnes of plastic waste annually [8] and contribute to the use and disposal hazardous chemicals [9]; and computational health research is associated with environmental harms related to the manufacture, use, and disposal of digital technologies. In fact, the digital sector (ICT) sector is estimated to account for between 2.1 and 3.9% of global greenhouse emissions, with predictions suggesting that this will increase as society moves towards digital solutions [10–12]; other digital technology related harms include those associated with mineral mining and the production of electronic (e-) hardware waste [13, 14]. Together, the health sectors’ associated environmental harms contribute to challenges for many communities in accessing clean air, safe drinking water, sufficient food, water, secure shelter (cities, settlements, infrastructure), and secure livelihoods (including fair employment and wages), all of which are determinants of both physical and mental health [15–19].

The adverse environmental and health impacts associated with the health sector produce an internal contradiction between the sector’s goal of improving health conditions, including health outcomes and equal access to healthcare, and the health risks associated with its environmental harms [20]. This is ethically problematic on a number of levels, of which three are noted. First, Pierce and Jameton (2004) draw on utilitarianism to argue that failing to consider the health burdens associated with the manufacture and disposal of healthcare related products creates imbalances in any utilitarian decision-making approach because it means ignoring key links in the consequentialist pathway that are associated with harms that come from the use of healthcare services. These scholars argue that when these health burdens are added, ‘everyday decisions unquestioned by ethicists and regarded as rational and even praiseworthy may be seen as questionable and possibly maleficent’ ([21], p. 119). As such, they say, it is important to categorise and include such harms in any decision-making process. Second, ignoring these health burdens goes against the ethical principles associated with planetary health, which promotes a holistic approach to health, emphasising the inter-relationships between health and the environment [22]. For planetary health, humans and the environment flourish together and a planetary health ethic requires that all aspects of environmental and human health are respected during decision-making practices. Finally, these health inequities create an environmental and health justice issue. Justice is a key underlying principle of many modern day societies, as well as a key underpinning of international biomedical, health research, public health, and environmental ethics frameworks [15, 23–26]. In a globalised world, to be just means ensuring the fair and equitable distribution of benefits and burdens not only within national boundaries but for all those who are subject to a given governance structure (cosmopolitan justice; [27]). Specifically, understandings of justice developed in recent decades argue that all individuals and communities affected by a particular process, technology and/or product wherever they are in the world, and whatever aspect of the product/process/technology they are affected by have moral standing and should be the subjects of justice considerations (see, for example, Marion Young [28] and Fraser’s, ‘all subjected principle’ [27]; for healthcare/research references, see [20, 29]). In the case of the environmental impacts associated with health research, a justice issue emerges because health/environmental benefits and burdens are unequally shared between communities with those who are most likely to be exposed to health harms being those least likely to benefit from the health research [20, 30].

Calls for an environmentally sustainable healthcare sector have spanned decades [24, 29, 31–34].^1^ Similarly–though to a lesser degree–calls have been made to address the environmental impacts of health research within the broader research context [13, 35–43]. While large strides are currently underway to address the environmental impacts of healthcare, particularly in developed countries whose healthcare systems contribute to a large proportion of national emissions–and much discussion has argued the importance of considering these issues either generally or explicity to address health inequities [20, 44–52]–, less is known about how health researchers in these countries are addressing these issues, if at all.

The aim of this paper is to present and discuss the findings of an interview study that explored UK data-intensive health researchers’ attitudes about the adverse environmental impacts associated with their practices, and how they view the importance of these considerations within their wider health research goals. The study is part of a broader project exploring ethical issues associated with the environmental impacts of UK data-intensive health related research. The research question was: what are the views and experiences of UK researchers working with data-intensive methodologies in the health arena about the need to consider the environmental impacts of their research? An interview methodology allowed in-depth exploration of researcher participants’ experiences and beliefs, as well as a detailed understanding of any underlying meanings and values.

Findings showed that health researchers wanted to address the adverse environmental and related health impacts of their research and they reflected on how they could consider these impacts in alignment with their own research goals. However, when tensions emerged, their own research topic was prioritised ahead of these concerns. This was related to their own desires as researchers and driven by the broader socio-political context of their research endeavours.

## Background: environmental and health harms associated with data-intensive methodologies

Data-intensive methods contribute to greenhouse gas emissions and other environmental impacts because of the energy and resources required to collect, store, and process data. In health research, data-intensive research practices include collecting and/or analysing vast swaths of clinical and associated data, such as electronic health records, imaging data, ‘omics data (proteomics, genomics, metabolomics etc), social media data, self-reported surveys, app-related data, and/or other passive data (location, sleep hours, tracker information) [53, 54]. These data consume huge data storage capacities, for example, a Californian health-based network with more than nine million members is estimated to have between 26–44 petabytes of patient data from electronic health records.^2^

Many large scale datasets are analysed using complex algorithms and/or artificial intelligence (AI) methods that also require large amounts of energy when used, including machine learning and natural language processing. For example, a recent study calculated the carbon emissions associated with the use of various algorithms in bioinformatics, and showed that the energy required to conduct a genome wide association study for just one disease trait to be equivalent to driving 100 km [14]. Furthermore, while efficiency gains are often touted as a way to mitigate energy increases associated with soaring data use, such arguments ignore rebound effects, that is, that efficiency gains rarely lead to decreases in consumption, and can sometimes lead to increased consumption because of behavioural and socio-political changes associated with efficiency gains [55].

Beyond emissions, the location of data centres and the cables that connect them can have impacts on the material environment (biodiversity, affecting the natural landscape). The use of unsustainable practices for extracting minerals for technological components and e-waste (discarded electronic appliances) disposal is also problematic [56, 57]. Mining-associated health and well-being harms are numerous [58] and include respiratory illness, injuries, cancers, and mental health. Community health risks occur through exposure to the air, water, soil, and noise pollution that come from mineral extraction and (highly toxic) processing and manufacturing [59, 60]. A survey 406 lower-to-middle income countries’ mining-related hazardous waste sites–affecting approximately 7.5 million people–showed that arsenic, lead, and mercury, all strongly associated with adverse health effects, contributed more than three quarters of the environmental risks at these sites [61]. Furthermore, only about one-fifth of e-wastes are formally collected and recycled globally, with most being dumped on landfills or traded illegally [57]. Resource recovery from e-waste landfills provides livelihoods and business opportunities, but unregulated recycling methods (open burning, incineration, acid stripping of metals) generate hazardous by-products which have been shown to be present at increased levels in individuals living around e-waste sites [62–65]. The WHO has stated that as many as 12.9 million women are working in the informal waste sector, and that ‘children are often engaged by parents or caregivers in e-waste recycling because their small hands are more dexterous than those of adults. Other children live, go to school and play near e-waste recycling centres where high levels of toxic chemicals, mostly lead and mercury, can damage their intellectual abilities’ [17].

Frameworks have been developed to help researchers using data-intensive methodologies mitigate the environmental impacts of their research. In the UK, for example, the *Digital Humanities Climate Coalition* has developed a toolkit to support humanities researchers consider the environmental impacts of their data-intensive practices.^3^ Lannelongue and colleagues (2021) have produced ten simple rules for making computing more environmentally sustainable in the life sciences. These include calculating the carbon footprint of research and considering this in a cost-benefit analysis; reflecting on where data is stored and its effect on energy consumption; and thinking about which hardware purchased, how it is recycled/re-purposed, and whether generated software can function on older hardware models. Addressing neuroscientists, Rae and colleagues (2022) have also called for researchers to consider how much data they collect, process and share. Despite these calls, little is known about if health researachers value the need to reduce the environmental impacts of their research, and how this relates to their broader research goals.

## Methodology

This study used an exploratory, qualitative, interview approach because it allowed for an in-depth exploration of researcher’ experiences and beliefs about their research practices and environmental issues, as well as any underlying meanings and values attached to these beliefs. Sampling was purposive: potential interviewees were selected based on their research into health-related topics using data-intensive methodologies. The goal was not to have a representative sample^4^ but rather to use the analysis of the interview data to generate hypotheses, which would then require further research to determine how representative they are for broader demographics.

### Recruitment

Potential participants were identified via a number of routes: (a) a list of publicly accessible successful applications to access the UK Biobank^5^ resource for which principle investigator researchers were based in the UK, had publicly accessible contact details, and whose profile related to the use of data-intensive research methods; (b) a publicly accessible list of individuals involved in all of the Genomics England^6^ clinical implementation partnerships whose profile related to the use of data-intensive research methods; (c) checking publications from various bioinformatics journals for UK-based authors working in data-intensive research including, for example, *Biodata and Mining*, and the *Journal of Biomedical Informatics*; (d) searching Web of Science using keywords associated with digital health research associated with biosensing ((“mobile sensing”; (“wearables” and “health”); (“biosensors” and “health and data”); (“digital phenotyping”); (e) various web searches for data-intensive health initiatives at various public institutions and organisations; and (f) snowballing. From this, a list of 145 relevant researchers and/or research consortiums were invited to participate. Following email invitations, 26 researchers agreed to participate. Interviews were conducted online or via phone between January and March 2022.

### Demographics

The sample comprised mainly of male participants (n = 21/26; in line with heavy bias in the field [66]). Interviewees were from a range of seniority levels (9 Professors; 13 research associates, fellows, lectures, or senior lecturers; one postgraduate (PhD) student; one health research data manager; and two from SMEs). Interviewees were from 13 different universities, and from a range of disciplines, including clinical research (n = 6), engineering (including AI)(n = 4), public health and/or epidemiology (n = 6), data science/bioinformatics (n = 6), health services research (n = 2), and data manager/curators (n = 2). (See Table 1.)

**Table 1:** Summary table of participant demographic information

Demographics	Number of participants
**GENDER**	
Male	21
Female	5
**SENIORITY / ROLE**	
Professor	9
Research associate, fellow, lecturer, senior lecturer	13
PhD student	1
Health research data manager	1
Small-to-medium enterprise (SME)	2
**DISCIPLINE**	
Clinical research	6
Engineering (including AI)	4
Public health/epidemiology	6
Data science/bioinformatics	6
Health services research	2
Data manager/curator	2

### Data collection and analysis

Interviews were conducted online or via phone and were digitally audio-recorded (except one interview, which was returned in written format). Interviews lasted between 25 and 65 min, with most (n = 16) being over 40 min. Interviews explored participants’ background, their use of data-intensive methods, and the type and quantity of data and methodologies used (including how data is accessed, collected, stored, and processed). They also explored participants’ understanding and views on issues around environmental sustainability for data-intensive practices, whether they viewed these concerns as relevant to their own practices, and if so, how they incorporated them (if at all) into their day-to-day decision-making (including any aspects associated with data collection, storage, processing; algorithm development). Knowledge of data storage locations and energy requirements was also explored, as was participants’ considerations of responsibilities associated with sustainability considerations, including trade-offs of values in practices. Throughout the interviews, participants were asked for examples of practices that were associated with their data-intensive methodologies and/or considerations of environmental sustainability.

Analysis of interview data was inductive using a version of thematic analysis that included two inter-linked rounds of coding: broad coding (memo-making and scanning interview transcripts for relevant themes), and detailed coding of the transcripts using NVivo software [67]. Coding was analysed and themes were developed. The theme relevant to this paper related to how values associated with environmental sustainability issues were incorporated (or not) in practice, and the tensions that emerged between this consideration and other research considerations.

Given the sample size, distinctions were not made between different interviewees’ disciplines or seniority position, and no differences were apparent during analysis in terms of their attitudes and experiences related to environmental considerations. This might be because interviewees were self-selected, and likely all were interested in sustainability issues. While this may bias the data, findings remain relevant as the aim of the study was not to make claims about representativeness, but rather was exploratory in nature, designed to generate hypotheses.

### Limitations

Low representation of women and those from the private sector is a limitation. Also, the sample is UK-based and therefore applies to the UK context of research culture, it also centres around data-intensive health research in the UK. Results will likely not be generalisable outside of the UK.

### Ethics

The study received ethics clearance from King’s College Research Ethics Committee (MRM-21/22-26574).

## Findings

Most participants were aware of the environmental and consequential health harms associated with their data-intensive methodologies, and while they rarely thought about them in their own practices, nor knew how to, they stressed the need to consider them. They noted challenges with doing this, while at the same time reflected on how some of their practices already aligned with such considerations. Despite this, tensions emerged with other research priorities, such as researchers’ personal drive to get a research result, their belief in the promise of data-intensive health research to improve health outcomes, and the competitive research environment in which they worked. Tensions were often resolved by de-prioritising environmental considerations.

### Considering the adverse environmental impacts of research

Participants expressed uncertainty about what it would mean to consider the adverse environmental impacts of their practices, though for most of them, consideration of research’s adverse environmental impacts was related to practices associated with decreasing resource and emission consumption of their own research: it was about *how* their research was being conducted rather than *what* was being researched.

At the same time, they were concerned about potential difficulties they envisaged could emerge when trying to address these impacts alongside other research priorities. Due to the varying levels of understanding about the issues, they used hypothetical examples to illustrate their concerns. Interviewee 23 understood how storing and processing data in colder climates could lead to a decrease in emissions associated with their data practices because less energy is required to cool the servers in these locations (servers become very hot when they are used and typically require air conditioning to cool them down). However, this interviewee struggled with the idea of balancing such concerns alongside other data storage values, such as data sovereignty (data sovereignty involves storing and processing data in the same country that it is collected to ensure benefit return to the country of origin -and in particular, the donating data participants). This interviewee reflected on this tension, highlighting that while they had not yet thought about this in their own practices, if the issue did emerge, it would require further consideration:

*you have got to have that data [the data that is being analysed] somewhere. And then there’s things about national and international sovereignty, holding the data, etc. It’s a whole territory that we’ve not really explored to much of a degree. [Alongside], let’s say, we want to have more efficient, we want to use computational systems where you know they don’t impact the environment. So that may be in colder parts of the world where they’ve got geothermal or renewable energy, that means that your data has to go there potentially. So, we have to think about things like that.*

Sometimes decisions about how to balance different values (economic, social, environmental) were taken out of researcher’s hands. Research funding bodies often needed to save costs irrespective of the environmental impact of such decisions, and researchers had little choice but to comply: *‘the funding body doesn’t put any stipulation around how and where you store the data…you always have to go with the cheapest price’* (interviewee 8). When researchers did have more control, many interviewees talked about other potential financial barriers to decreasing the resource and emission consumption of their data-intensive practices. A number of researchers were working on complex projects for which they were unsure how to address the environmental impacts of their research. They sensed that outside (costed) expertise could help them, but that there was limited (or no) funding for this. In the below extract, interviewee 22 describes how funding bodies only allow researchers to claim a certain budget for each research proposal, and because this budget is small it needs to be allocated to the primary aim of the research project and any mandatory criteria (e.g., data governance, ethics etc.). This meant there was no spare budget for costing expertise and environmental sustainability know-how to understand how best their project could consider its adverse environmental impacts. This interviewee described their difficult position–they were unable to conduct their own research (what the interviewee refers to as ‘technical’ project in the extract below) if they wanted to assess the adverse environmental impacts of their work (the ‘social science’ project in the extract below):

*If I write a grant…all these other aspects [like]… sustainability, that requires a very complex set of skills…[.]… I only get two [full time equivalents] for the whole grant…So when you put that into context, is this important yes, but…either I deliver a social science project or I deliver a technical project, [you can’t do] both…we all realise the importance of this. In the end we are citizens, right. But those citizens like me that also are scientists in technology areas….we have complex problems and a very isolated approach to resource our problems and the research to address those.*

Beyond financial cost, time was also an important constraint. With so many data governance requirements attached to the collection, storage, and processing of data, interviewees sensed little time remaining to prioritise practices associated with decreasing resource and emission consumption. Participants only had a basic understanding of the issues and struggled with how they could better acquaint themselves with their time often at a minimum. These constraints were especially pertinent in contexts such as working in/with the private sector. Interviewee 15, who was an academic researcher, used the example of academic-industry partnerships to emphasise how, when working with industry, fast-paced decision-making was required, meaning that environmental factors could be ‘part of the conversation’, but nothing more than low on their research agenda, and as such, they had little opportunity nor time to think about them:

*people are worried about air conditioning, overheating, polluting data, governance, you know, those types of really mundane, day-to-day things which threaten datasets….when everybody’s strung out and worried about keeping things going, to then have an additional layer of concern….So it is definitely there, definitely part of conversation. But….people need their data and they need to get it secure. So getting through your audits and making sure the governance is okay is sometimes higher up the profile*.

### Reducing resource and emission consumption: aligning with current practices

Despite the perceived constraints participants associated with reducing their emissions and resource consumption, during interviews, and when asked about whether they considered the environmental impacts of their research within their practices, many participants reflected–often for the first time–on how some of the research practices that they valued already aligned with these goals. This alignment was most evident in their discussions related to open science and reducing the financial cost of their research. Taking each in turn, those participants supporting open science initiatives spoke about the importance of promoting openness and reproducibility of their data and algorithms. The intersection of these practices with environmental sustainability was not something interviewees had previously considered, but during the course of interviews, participants reflected on the synergies between the goals of open science and the broader goals of environmental sustainability:

*[open science and data sharing is about] whatever we produce is more likely to be adopted and to last. In [that] sense…we could argue that our output is particularly environmentally friendly…but it’s not something that we’ve ever given any thought to* (interviewee 21).

Second, nearly all interviewees explained how a key aspect of their role was to reduce their research’s financial energy and resource costs. This was mainly achieved through efficiency gains in algorithmic processing. Efficiency-dependent reductions were perceived to go ‘hand in hand’ (interviewee 1) with environmental sustainability concerns: ‘*sustainability… I guess [is] efficiency’* (interviewee 8). Interviewee 21, who had not previously considered the relationship between efficiency and reducing the environmental impacts of their work, reflected on how their optimising of code, as well as the desire to converge their algorithms as quickly as possible,^7^ was intricately tied to consuming less energy, and therefore had positive environmental impacts: ‘*we want models to converge as fast as possible. So, if it’s running for five minutes rather than five hours, my assumption is it’s consuming less energy’* (interviewee 21). Interviewee 9 similarly explained how decision-making associated with data platform choice, while based on financial factors, also closely aligned to environmental gains: *‘power consumption costs were something we considered [when deciding whether to run algorithms in the cloud] and I guess that maybe… I can’t remember anyone explicitly mentioning environmental issues, but it’s closely related’*. Likewise, interviewee 24 explained how their exploration of ways to minimise expensive mistakes connected to leaving an algorithm running for too long was also tied to environmental gains associated with reducing energy consumption:

*we’ve got a problem in that if I create a very expensive virtual machine and leave it running for a year without ever switching it off…we are trying to find ways of tagging and labelling things that we’re creating, and reporting on their cost because this does concern us that this [the algorithm continuously running and becoming financially costly] might happen.*

Interviewees therefore realised that financial and environmental impacts were aligned, meaning that they were being more environmentally sustainable than they thought. As seen above in the context of open science, this alignment was perceived to offer an approach to consider the adverse environmental and consequential health impacts of their research in practice. This was despite many interviewees explicitly stating that the environmental benefits of these approaches were not considered during decision-making: ‘*I think of the efficiency of a job [when developing algorithmic code]…but more because of the time it will take me to run a job, not because of the environmental impact’* (interviewee 14).

### De-prioritisation of environmental concerns

Not all practices that researchers valued aligned with environmental goals. For these other practices, when tensions emerged, environmental concerns were rarely given primacy. Rather, the need to complete the research in the quickest way possible was given priority. This was not only because of competitive research environments but also because of the belief that their research would have real health benefit; researchers viewed data-intensive research methodologies as key to addressing many poor health related outcomes. In the below, these priorities are described under the sub-headings: goal-driven health research and competitive research cultures; health as the ultimate value; and the need for ever more data consumption to address health issues.

### Goal-driven health research and competitive research cultures

Participants spoke about the importance of getting a research ‘result’ (interviewee 10). Competition was constructed in terms of a ‘publish or perish’ discourse, i.e., the need to publish research findings to secure academic research status and further research funding [68]. Interviewee 5 explained how when UK Biobank (the database they were accessing for their research) releases new data, research laboratories around the world ‘jump on it straight away’ to ‘run every possible association’ so that they can publish the findings immediately. This was despite whether such association studies would be valuable to other health researchers. In fact, this interviewee questioned the necessity of running many of these association models without being framed in terms of having a specific research question: ‘*I think there’s a case of people wanting to get there first and so will just perhaps do more than is necessarily needed or wanted in order to do that’.*

This publish or peril mentality often became prioritised at the expense of all else: *‘it’s more about trying to solve the problem….by whatever means, and not necessarily thinking much about the consequences [including adverse environmental impacts]’* (interviewee 18). Results were valued to such an extent, explained interviewee 10, that there was little reason for researchers to consider resource or emissions reductions, as doing so was unlikely to affect publishing papers-a mark of value for the individual researcher: ‘*why bother doing a good implementation [in terms of mitigating environmental impacts of the research] when the result will be the same and it’s going to lead to the same paper’.* Furthermore, a culture that warranted results at any cost provided a difficult context for researchers who, despite this, still wanted to reduce their consumption.

### Health as the ultimate value

Achieving a research result was not just perceived as vital for competitive reasons but was also perceived important because it would lead to positive health outcomes. Participants discussed their desire to help patients (*‘the end gain for the patients, that’s really always where we’re focused on’* (interviewee 13)). This desire was so strong that decreasing the resource and emission consumption of a research project was sometimes (or often) de-prioritised. In the extreme position, of which examples were provided by only a few interviewees, interviewee 13 described how researchers were so focussed on the goals of their research, that they became ‘blinkered’ about the value of their research more broadly. For them, the value of getting their research completed so that it could solve a health problem was so paramount that when in jeopardy, any resource and/or emission consumption was justified no matter the environmental or health cost, including last minute flying and/or ordering new digital devices:

*probably health data scientists, they’re kind of blinkered on the idea of like you’re doing good because you’re trying to solve such an existential problem of healthcare, you just think I’ll do whatever it takes to get that done….There’s a lot of downstream costs…of ordering things last minute…or you go fly somewhere that you need to go and speak to some collaborators…or buying devices that you just need to transfer data onto quickly. There’s a…flippant side of that but it’s just because it’s serving the higher purpose of trying to do this research quicker.*

In a less extreme but more prominent view, interviewees pondered the importance of considering the environmental impacts associated with their research, but overall believed that there was a net ‘positive effect’ of their research compared to the more negative effects associated with its environmental impacts. Interviewee 18 analogised this to testing during the Covid-19 pandemic, when the health of those who could access a Covid-test was prioritised compared to the environmental- and health-related risks that were associated with the plastic waste associated with this testing:

*I think there’s a really interesting philosophical point isn’t there about, in health care, because it’s about the primacy of human life and protecting that, isn’t it? And we can see it with all the Covid testing, can’t we the amount of plastic waste that’s come up for the sake of protecting people against Covid. And it seems that health care, for people suddenly outweighs sustainability of the planet.*

### The need for ever more data consumption to address health issues

While participants seemed at ease contemplating the need to reduce resources and emissions associated with their research practices, they viewed their data-intensive methods as being necessary to answer ever more difficult health questions morally relevant to society and therefore were in support of the continual and increased consumption of data storage and/or processing power. For example, interviewee 1 described the imperative of developing continuous quantification for all aspects of people’s molecular mechanisms as a way in which understanding of health, ageing and the prevention of disease could be improved: *‘if you just do all the molecular readouts of people all the time, then you can build models of what keeps people alive, in terms of keeping them healthy. So, then you can fund for healthy ageing, disease prevention, so, it goes far beyond what we currently do*’. Such beliefs led researchers to drive the urgency for both ever-increasing datasets, as well as more powerful algorithms for analysis, thereby increasing consumption. Interviewees explained how they were quickly running out of computational space to run their algorithms (*‘I need larger memory….we developed a very large model. But even this model cannot fit on our own GPU computer’* (interviewee 4)); they were also in need of more data to run their algorithms: *‘the algorithms are getting more and more advanced….Our data will not catch up with the power of the algorithm’* (interviewee 11). Only a few interviewees reflected on this need to collect ever more data, raising concerns about whether this was always the most appropriate approach for addressing health problems. For example, interviewee 2 reflected on the fact that often health researchers default to use machine learning when less energy-intensive algorithms may be more suited to address a health concern: *‘I think that’s something that probably machine learning research don’t do often enough. It’s like go back to more traditional methods or algorithms or models that are much less compute heavy. And that maybe, depending on the task, could be fully appropriate to addressing the problem’.*

## Discussion

Most participants emphasised the importance of considering the adverse environmental and consequential health impacts of their research practices as long as it did not constrain the type of research they were conducting. However, they lacked understanding of how to address these issues in practice and pointed to various resource and time constraints associated with the broader research culture that prevented them from considering these issues. Addressing the adverse environmental impacts of their research in-practice through the incidental alignment with other research priorities was seen as a way forward: rather than setting out to mitigate the environmental costs of their research, participants pointed to the convenient happenstance between, on the one side, some of the current ways they valued conducting research and, on the other hand, environmental considerations. Such alignments are well-established at promoting environmental considerations [69–72], and can be effective at placing environmental issues on an agenda in a non-threatening way because other agendas have more visibility or buy-in and can provide a basis to endorse environmental considerations. In fact, open science has been promoted as a key aspect of environmentally sustainable research because of shared goals associated with minimising waste and increasing the reproducibility of science [73, 74].

Nevertheless, the convenient happenstance between values did not always occur, and, as has been seen in the literature more broadly, tensions emerged between environmental considerations and other research priorities [39, 70, 71, 75–80]. When this happened, valuing environmental considerations and their consequential detrimental health impacts was often de-prioritised. This was because (most) participants viewed their data-intensive research as bringing health benefit to particular members of society, so much so that they positioned their work as valuable and justified at all costs. This paper’s central claim is that these beliefs, and the implicit justifications participants had about their research, are ethically problematic for two main reasons, which we map out below.

First, while health research can improve health outcomes, conducting health research without due consideration for its associated environmental and health harms means ignoring health harms for some, while prioritising health benefits for others. As discussed in the introduction, this becomes a justice concern because considering justice issues requires the balancing of health burdens and health benefits for *all of those subjected* to a particular process and or governance framework (a cosmopolitan approach). This includes those who are associated with the manufacture, use, and disposal of digital technologies related to data-driven health research as well as those who benefit from this research, regardless of where individuals experiencing health harms and benefits reside in the world [27]. While questions arise about how to balance justice issues that affect both citizens and services within a particular country, as well as those who are outside of nations, Brock (2015) explains that these two requirements are not mutually exclusive and that a compromise can provide adequate space for both. Using the case study of the UK health service, she argues that those within a state should receive special attention but that we still have a moral obligation to make low or reasonable modifications to our own governance structures because of the negative duty to refrain from harming [81]. We consistently see this playing out outside of health, for example, when conscious consumer movements and NGO (non-governmental organisation) pressures have driven improvements of social, economic and health conditions for those involved in the manufacture and development of consumer products [28]. Unfortunately, oftentimes, this does not happen in research ethics practice. While research ethics frameworks do not distinguish their considerations of research-related harms/benefits at national and international levels, they do limit harm considerations to those individuals and/or communities who are directly affected by the research–individuals/communities who often reside within a particular nation. At the same time, research ethics considerations often adopt a more ‘cosmopolitan’ way of considering research benefits, which includes taking into account individuals/communities who reside anywhere in the world. The result is an un-balanced cost-benefit ratio with respect to research harms and benefits–something that needs to be addressed.

Second, data-intensive health research might not always be the only or most appropriate way to improve the health of those in society–something that was noted by at least a few of the interviewees. There is already ample evidence that by far the greatest population health benefits come from improving the social determinants of health.^8^ Yet for many of the interviewees, positive health outcomes were synonymous with the collection and analysis of more data for their own research endeavours. This belief seemed to be compounded by the techno-solutionist assumption that data-intensive health research was the best way to address societies health problems [82], leaving little room for discussing how we address the ever increasing consumption of resources through the collection and analysis of evermore data [55]–something that is part of the UK’s (and other developed countries’) broader consumptive and extractive practices. This was also compounded by the goal-driven nature of the research and the neo-liberal context of research culture, that placed value on individualism, entrepreneurialism, productivity, and competition [39, 80, 83–85]. As such, while, as argued above, and argued elsewhere, health researchers should expand their moral gaze to consider the environmental harms associated with their research practices [20], this raised tensions with other research priorities.

### Moving forward: social-political culture changes

While the findings of this study are preliminary and from one sector of health research in the UK, they are still instructive. They suggest that while it is important to consider the health/environmental inequities associated with health research, it is not feasible to place such a responsibility (solely) on researchers themselves. Researchers are working in a socio-political context that makes it difficult for them to respond to such justice concerns (*forthcoming*). As such, addressing these concerns needs to come from changes in research culture beyond (or at least in addition to) those at the level of individual researchers, such as within the sector itself. At the same time, research culture and practice are embedded in wider socio-political contexts of consumption and competition that are also difficult to change at the sector level.

Nevertheless, there are various ways in which the wider research sector could support the health research community address its associated health and environmental concerns. Several ways of moving forward are noted below. Before noting these points, it must be stressed that these practices alone will not address the environmental and health injustices that arise as a consequence of the research process. It must also be emphasised that these practices must not lead to individualising at the level of the researcher, what is a societal problem. Nonetheless, if changes in practice are implemented in a careful way–that is, not via compliance, but by building a shared understanding of the importance of expanding the moral gaze to include such issues–we can build motivation to make changes (and amplify existing motivation that is already present (*forthcoming*))–that will at the very least, create a legacy for thinking about these issues.

First, funders could value environmental considerations and the consequential detrimental health harms associated with research by ring-fenced funding and resources for researchers to implement more environmentally sustainable processes within their research practices. This could occur during grant application processes, in which, for instance, funding bodies allow researchers to budget to store and process their data in more expensive but more environmentally sustainable data centres whose management also consider issues of social justice, such as labour arrangements and so forth. Or it could be in a form similar to that provided by, for example, the UK National Institute of Health Research, where researchers can apply for top-up funding to make their already awarded research more environmentally sustainable [86]. Two research participants in our study were aware of such funding, but it is unknown how regularly this funding is used more widely.

More broadly, funding bodies and/or research institutions could value environmental considerations and its impact on health inequities by providing expertise for researchers on how to reduce the adverse environmental and health impacts of their research, so that researchers could draw upon this while developing their research proposals. This was provided by the Arts Council England during the implementation of their environmental agenda that required funding applications to consider these issues.^9^ In addition, research ethics committees could play a role in helping researchers consider how generated study data will be stored and/or processed. At present, research ethics committees lack expertise in this area and are over-burdened [87]; training could be made available to committee members in this area or the composition of such committees could be updated to allow for members to meaningfully review relevant applications. Finally, institutions could provide academic recognition to include more than just conducting health research; but to also include *how* the research is conducted. In the last decade, we already see a move in the research agenda, at least in the UK, to including equity, diversity, and inclusion considerations, and this could be extended to also reflect on environmental considerations. We have also seen the research agenda move to recognise the importance of research’s societal impact [88] and this, too, provides a precedent for expanding academic recognition beyond the reporting of research findings in academic journals.

Overall, as noted above, change needs to happen at the socio-political level, but small changes in research practice and culture can help promote a wide moral gaze that builds awareness and reflects on the environmental and health harms associated with research practice, not just for the here and now, but also to the health of generations beyond.

## Conclusion

This study explored how UK health researchers using data-intensive methodologies consider the environmental impacts of their research practices, and how they manage these alongside other research priorities. It shows that while researchers wanted to expand their moral gaze to consider the environmental and health harms associated with their research, and were doing so in some instances, these issues are often de-prioritised because of tensions associated with broader research culture. It has suggested various ways the sector can begin to change research culture, but ultimately notes that the socio-political climate in which research is embedded can make this difficult to achieve.

### Electronic supplementary material

Below is the link to the electronic supplementary material.

Supplementary Material 1

## Data Availability

The datasets used and/or analysed during the current study are available from the corresponding author on reasonable request and in line with consent requirements.

## Footnotes

1 Also see https://noharm.org/.

2 And potentially much more as further records are digitalised, and the propensity for data grows. cited in: Managing the healthcare information stream, Commvault. 2015. chrome-extension://efaidnbmnnnibpcajpcglclefindmkaj/http://webdocs.commvault.com/assets/managing-the-healthcare-information-stream.pdf.

3 https://www.cdcs.ed.ac.uk/digital-humanities-climate-coalition.

4 A representative sample would have required a survey style methodology, which would not have allowed for an in-depth exploration of participant views and beliefs.

5 UK Biobank is national UK population biobank that stores samples and associated data from over 500,000 UK participants.

6 Genomics England is a UK project that sequenced 100,000 genomes from patients who had a rare disease or cancer for analysis for both research and clinical care.

7 i.e. develop algorithms that no longer need to be trained.

8 https://www.who.int/health-topics/social-determinants-of-health#tab=tab_1.

9 For example, see https://www.artscouncil.org.uk/creative-matters/news/season-change-toolkit.
