# Supplementary material for: UK health researchers’ considerations of the environmental impacts of their data-intensive practices and its relevance to health inequities
Source: BMC Med Ethics. 2023 Oct 27;24:90. doi: 10.1186/s12910-023-00973-2 (PMC10612270; doi:10.1186/s12910-023-00973-2)
Supplement: Supplementary file 1 — Supplementary Material 1 [file 12910_2023_973_MOESM1_ESM.docx]

**INTERVIEW SCHEDULE**

**About the interview**

- Provide introduction to the project and myself
- **Ethics.**Ensure I have a signed consent form before beginning and that all questions are answered.
- **Anonymity.** Remind interviewees that all quotes will be de-identified.
- **Interview style.** I’ll be asking some questions to which the answer may seem obvious, but we’re doing it because we want to understand your perspective without making assumptions.
- **Recording.** I will be recording the interview as long as you have provided consent. If not, I will be taking notes of our conversation.
- **Answering questions.** You can decline to answer questions and stop the interview at any time should you wish.
- **Ask if they have any questions for me:** both at the beginning and end of interview.
- **Interview schedule.** Questions do not necessarily need to be asked in order or with the exact wording, as long as the main point associated with each question is asked. Questions are standard text. Optional prompts are in italics as sub-questions/comments.

**Participant’s background**

1. What discipline (or disciplines) do you see yourself as belonging to and what are researchers in your field generally doing/trying to achieve in this discipline?
2. How does your particular research fit into this? If you have a number of interests or research areas, please describe all of them.
3. Reflecting on your own research, what data and methodologies do you use in your research and/or what concepts do you apply (depending on the researcher we speak to)(please describe methods used in *each* of your research areas)?
   1. *What are the main findings of your research/research areas in lay terms (i.e. how do you see your research as contributing to your research field)*
   2. *What do you think your research will achieve in the long-term (what do you see as the benefit of your research?)*
4. Can you describe to me with examples any challenges you have come across along the way of your research in terms of your methodologies or your findings (depending on what is more relevant to them)?
   1. *How have you tried to address them?*

**Questions specifically about your practices when it comes to data**

1. Could you describe, in as much detail as possible, which data you specifically use and the size of the dataset you use during your research?
2. Could you describe, in as much detail as possible, how you access the data you use during your research?
   1. *Cloud or downloaded, and their views on this*
   2. *Collected or secondary analysis*
3. How are the data analyses you conduct powered?
   1. *Do you know how much it costs? What energy provider is used? If not, why not?*
4. What practices and processes do you have in place for the end-of-life of your computers in terms of recycling and/or repurposing (and if you work in a lab, then any other waste too)

**Sustainability / environmental impacts**

1. How, if at all, do you understand the term environmental sustainability? Feel free to talk as broadly or as narrowly as you like?
   1. *What does sustainability mean to you? What how is sustainability understood in your discipline?*
2. What do you see as the relationship between environmental and other forms of sustainability, if any?
3. Reflecting on your own research, how, if at all, do you see your research as relating to the issue of environmental sustainability?
   1. *Related to data at all?*
   2. *How much of an issue do you think the adverse environmental impacts of data-intensive research are for researchers using data-intensive methods?*
4. What are your views about the need to consider aspects of environmental sustainability (the environmental impacts of your research) in your research practice?
   1. *How high on the agenda is it?*
   2. *Is sustainability something that your colleagues are thinking about?*
5. Do you feel you have the skills to address the environmental impacts of your research (if they view this as important), and if so, could you describe what these skills are?
6. Who has the responsibility to ensure that those developing algorithms consider the adverse environmental impacts associated with their work, and why do you think this?
   1. *Do you think sustainability is important in your disciplines/profession/research – how seriously is it taken?*
   2. *What incentives are there in your discipline for considering these issues – and where from?*
7. How should trade-offs (be) managed between the environmental cost of storing and processing data and other benefits of conducting research, if at all? How could this be improved?
   1. *Give examples of instances in which you have had to consider this, or if not this, other trade-offs*
8. What guidelines or initiatives are you aware of pertaining to the environmental impacts of research in the health/data field or other research fields?
   1. *What do these guidelines say?*
